# Amyloid-β and tau deposition in traumatic brain injury: a study of Vietnam War veterans

**DOI:** 10.1093/braincomms/fcaf009

**Published:** 2025-01-10

**Authors:** Hannah de Bruin, Colin Groot, Suzie Kamps, Everard G B Vijverberg, Anna Steward, Amir Dehsarvi, Yolande A L Pijnenburg, Rik Ossenkoppele, Nicolai Franzmeier

**Affiliations:** Alzheimer Center Amsterdam, Neurology, Vrije Universiteit Amsterdam, Amsterdam UMC location VUmc, Amsterdam 1081 HZ, The Netherlands; Amsterdam Neuroscience, Neurodegeneration, Amsterdam UMC, Amsterdam 1081 HV, The Netherlands; Institute for Stroke and Dementia Research, University Hospital, Ludwig Maximilian University of Munich, Munich 81377, Germany; Alzheimer Center Amsterdam, Neurology, Vrije Universiteit Amsterdam, Amsterdam UMC location VUmc, Amsterdam 1081 HZ, The Netherlands; Amsterdam Neuroscience, Neurodegeneration, Amsterdam UMC, Amsterdam 1081 HV, The Netherlands; Alzheimer Center Amsterdam, Neurology, Vrije Universiteit Amsterdam, Amsterdam UMC location VUmc, Amsterdam 1081 HZ, The Netherlands; Amsterdam Neuroscience, Neurodegeneration, Amsterdam UMC, Amsterdam 1081 HV, The Netherlands; Alzheimer Center Amsterdam, Neurology, Vrije Universiteit Amsterdam, Amsterdam UMC location VUmc, Amsterdam 1081 HZ, The Netherlands; Amsterdam Neuroscience, Neurodegeneration, Amsterdam UMC, Amsterdam 1081 HV, The Netherlands; Institute for Stroke and Dementia Research, University Hospital, Ludwig Maximilian University of Munich, Munich 81377, Germany; Institute for Stroke and Dementia Research, University Hospital, Ludwig Maximilian University of Munich, Munich 81377, Germany; Alzheimer Center Amsterdam, Neurology, Vrije Universiteit Amsterdam, Amsterdam UMC location VUmc, Amsterdam 1081 HZ, The Netherlands; Amsterdam Neuroscience, Neurodegeneration, Amsterdam UMC, Amsterdam 1081 HV, The Netherlands; Alzheimer Center Amsterdam, Neurology, Vrije Universiteit Amsterdam, Amsterdam UMC location VUmc, Amsterdam 1081 HZ, The Netherlands; Amsterdam Neuroscience, Neurodegeneration, Amsterdam UMC, Amsterdam 1081 HV, The Netherlands; Clinical Memory Research Unit, Lund University, Lund 221 00, Sweden; Institute for Stroke and Dementia Research, University Hospital, Ludwig Maximilian University of Munich, Munich 81377, Germany; The Sahlgrenska Academy, Institute of Neuroscience and Physiology, Psychiatry and Neurochemistry, University of Gothenburg, Gothenburg 413 45, Sweden; Munich Cluster for Systems Neurology (SyNergy), University Hospital, Ludwig Maximilian University of Munich, Munich 81377, Germany

**Keywords:** traumatic brain injury, war veterans, amyloid-β, tau, PET

## Abstract

Traumatic brain injury is widely viewed as a risk factor for dementia, but the biological mechanisms underlying this association are still unclear. In previous studies, traumatic brain injury has been associated with the hallmark pathologies of Alzheimer’s disease, i.e. amyloid-β plaques and neurofibrillary tangles comprised of hyperphosphorylated tau. Depending on the type and location of trauma, traumatic brain injury can induce spatially heterogeneous brain lesions that may pre-dispose for the development of Alzheimer’s disease pathology in aging. Therefore, we hypothesized that a history of traumatic brain injury may be related to spatially heterogeneous amyloid-β and tau pathology patterns that deviate from the stereotypical temporo-parietal patterns in Alzheimer’s disease. To test this, we included 103 Vietnam War veterans of whom 65 had experienced traumatic brain injury (*n* = 40, 38.8% mild; *n* = 25, 24.3% moderate/severe). Most individuals had a history of 1 (*n* = 35, 53.8%) or 2 (*n* = 15, 23.1%) traumatic brain injury events. We included the group without a history of traumatic brain injury (*n* = 38, 36.9%) as controls. The majority was cognitively normal (*n* = 80, 77.7%), while a subset had mild cognitive impairment (*n* = 23, 22.3%). All participants underwent [^18^F]florbetapir/Amyvid amyloid-β PET and [^18^F]flortaucipir/Tauvid tau-PET 39.63 ± 18.39 years after their last traumatic brain injury event. We found no differences in global amyloid-β and tau-PET levels between groups, suggesting that a history of traumatic brain injury does not pre-dispose to accumulate amyloid-β or tau pathology in general. However, we found that traumatic brain injury was associated with altered spatial patterns of amyloid-β and tau, with relatively greater deposition in fronto-parietal brain regions. These regions are prone to damage in traumatic brain injury, while they are typically only affected in later stages of Alzheimer’s disease. Moreover, in our traumatic brain injury groups, the association between amyloid-β and tau was reduced in Alzheimer-typical temporal regions but increased in frontal regions that are commonly associated with traumatic brain injury. Altogether, while acknowledging the relatively small sample size and generally low levels of Alzheimer’s disease pathology in this sample, our findings suggest that traumatic brain injury induces spatial patterns of amyloid-β and tau that differ from patterns observed in typical Alzheimer’s disease. Furthermore, traumatic brain injury may be associated with a de-coupling of amyloid-β and tau in regions vulnerable in Alzheimer’s disease. These findings indicate that focal brain damage in early/mid-life may change neurodegenerative trajectories in late-life.

## Teaser

De Bruin *et al*. report that traumatic brain injury does not inherently elevate the Alzheimer’s pathologies amyloid-β or tau but is associated with their unique spreading patterns and interactions. Traumatic brain injury shows early fronto-parietal lobe involvement—regions affected only later in Alzheimer’s disease—indicating it may influence neurodegenerative pathways.

## Graphical Abstract

**Graphical Abstract:**
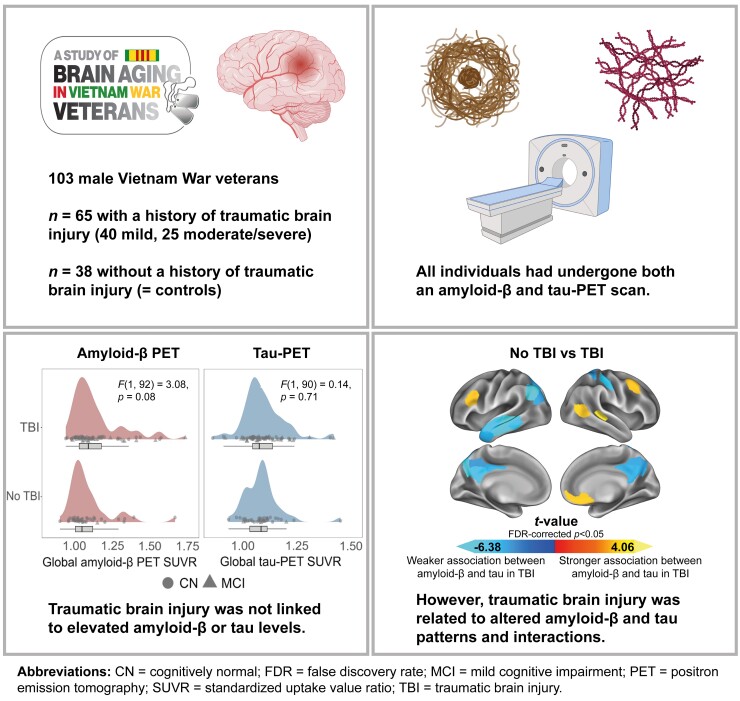

## Introduction

Although the role of traumatic brain injury (TBI) as a risk factor for developing dementia is well established, the biological mechanisms underlying this association remain elusive.^1-10^ Amyloid-β (Aβ) and hyperphosphorylated tau are the two hallmark pathological proteins of Alzheimer’s disease^11^—the most common cause of dementia^12^—and recent studies have explored their potential role as missing pathophysiological link(s) between TBI and cognitive decline.^13^ However, the current literature presents inconsistent findings, with some studies demonstrating a clear increase in Aβ-PET and tau-PET signals in TBI, while others reported no effects.^13^

Given the strong association of tau pathology with neurodegeneration and cognitive decline,^14,15^ it is key to gain a better understanding of the factors driving distinct patterns of tau spread. In line with previous preclinical *in vivo* and *in vitro* studies,^16-18^ recent human neuroimaging studies indicated that tau propagates throughout the brain following the functional connections from the putative sites of tau onset (i.e. tau epicenters).^19,20^ In Alzheimer’s disease, when Aβ plaques are widely present across the neocortex, Aβ is thought to drive tau outside of the initial site of tau deposition to gradually spread to connected regions.^16,19-23^

In aging populations, the initial site of tau deposition is typically located within the (trans)entorhinal cortex.^16,21^ However, recent studies in Alzheimer’s disease have shown substantial inter-individual heterogeneity in tau spreading patterns mirroring clinical symptoms,^24-31^ presumably originating from varying tau epicenters.^19^ In contrast to tau, the spreading patterns of Aβ are more diffuse and tend to localize first in regions with a high metabolic demand (i.e. hub regions), irrespective of the clinical phenotype.^32-34^ Unsurprisingly, the relationship between Aβ and tau depends on their respective locations in the brain,^34^ and—given the association of both Aβ and tau with neural networks—this relationship is likely to be more pronounced in hub regions compared with non-hub regions.

Interestingly, animal models have shown that mice exposed to TBI generally accumulated Aβ and tau close to the area of injury,^35-37^ and in one specific study, the authors showed that p-tau spread from this initial site of injury to synaptically connected regions.^35^ Given the diverse events that can cause TBI, which can in turn result in both focal brain damage and diffuse axonal injury,^38^ there might be substantial heterogeneity in the initial site of protein deposition. Consequently, this may lead to variations in the relationship between Aβ and tau spreading patterns. Hence, a major open question is whether TBI is associated with global increases as well as spatial variability in the distribution of Alzheimer’s disease pathology across the brain. To investigate this, we included 103 Vietnam War veterans of which 65 experienced TBI. Our goals for the current study were to (i) assess whether a history of TBI was associated with elevated global or regional Aβ- or tau-PET signal, (ii) establish whether a history of TBI is associated with changes in regional Aβ- or tau-PET patterns while accounting for the global Aβ- or tau-PET signal, (iii) investigate the spatial heterogeneity of tau epicenters in TBI, and (iv) assess the moderating effects of TBI status on the association between Aβ- and tau-PET.

## Materials and methods

### Participants

We included 103 male Vietnam War veterans from the Alzheimer's Disease Neuroimaging Initiative Department of Defense (ADNI DOD) study. Inclusion was based on the availability of at least one Aβ- ([^18^F]florbetapir/Amyvid) and tau-PET ([^18^F]flortaucipir/Tauvid) scan as well as data on TBI history and associated symptoms. Only individuals with a history of non-penetrating TBI were included (this information was either available or penetrating damage was checked on MRI). Since only two females remained after these initial inclusion criteria, we decided to only include male individuals to increase homogeneity of the study sample. The total sample included 80 individuals with normal cognition and 23 individuals with mild cognitive impairment.^39^ All study procedures were conducted in accordance with the declaration of Helsinki, ethical approval was obtained by ADNI investigators. All study participants provided written informed consent.

### TBI classification

Individuals were asked whether they had experienced head or neck injuries before, during or since the Vietnam War, or in the past year. They were told this could include an injury from a car/jeep/bicycle/helicopter or some other moving vehicle accident, a fall or from being hit by something (e.g. a fall from a bike or hit by a rock), being hit or violently shaken by someone, having been nearby when an explosion or a blast occurred, or being choked. TBI information was only included when the TBI event happened prior to the Aβ- or tau-PET scan. We compared the reported year, age and symptoms of TBI events with the specific time period in which each TBI event was reported to have occurred (before/during/since the Vietnam War, or in the past year), and in case of discrepancies (e.g. age, year and symptoms of a TBI event were reported, but no specific time period of the TBI was defined; or the reported time period did not match the age and year of the TBI event), we made adjustments to ensure consistency and accuracy in our data analysis. TBI history was dichotomized as ‘yes’ in case individuals experienced at least one TBI event in the given time periods, otherwise it was set to ‘no’. TBI symptom severity was based on the criteria of the Department of Defense and Veterans Affairs guideline to classify head injury severity.^40^ We selected this guideline as it is evidence-based, developed by a multidisciplinary team of experts, straightforward, and well-suited for military and veteran populations. Available criteria in the ADNI DOD data were loss of consciousness (n/a, 0–30 min mild, >30 min and <24 h moderate, >24 h severe), alteration of consciousness (feeling foggy, confused, disoriented, dazed, in a stupor, or ‘seeing stars’; n/a, <24 h mild, >24 h moderate/severe), and amnesia (n/a, <24 h mild, >24 h moderate/severe). Based on this, overall TBI severity was set to moderate/severe TBI when ≥1 of the symptoms fell into the moderate/severe category, otherwise it was set to mild TBI. This led to the final TBI status of no TBI, mild TBI or moderate/severe TBI. TBI frequency was computed by summing the TBI events of all time periods.

### MRI and PET acquisition and pre-processing

Each participant completed an [^18^F]florbetapir/Amyvid Aβ-PET scan and an [^18^F]flortaucipir/Tauvid tau-PET scan (detailed acquisition methods can be found at https://adni.loni.usc.edu/study-design/collaborative-studies/dod-adni/). For PET imaging, data collection occurred after intravenous administration of ^18^F-labeled tracers, with Flortaucipir data gathered over six 5-minute frames between 75 and 105 min after injection, and Florbetapir data over four 5-minute frames from 50 to 70 min after injection (additional details are available at http://adni.loni.usc.edu/methods/pet-analysis-method/pet-analysis/).

All imaging data were inspected for artifacts before preprocessing the data. For structural MRI, T1-weighted images were bias-corrected, segmented, and non-linearly warped into Montreal Neurological Institute (MNI) space via the CAT12 toolbox (https://neuro-jena.github.io/cat12-help/). Dynamic PET scans were realigned and averaged to produce single Flortaucipir/Florbetapir images, which were then rigidly aligned with the T1-weighted MRI. The inferior cerebellar grey and the whole cerebellum were defined as reference regions for Flortaucipir and Florbetapir, respectively.^41^ We used the cortical Schaefer atlas with 200 regions of interest (ROIs) for regional analyses. Using the non-linear normalization parameters from CAT12, our reference regions and the 200 Schaefer ROIs were warped from MNI to the T1-native space. Then they were masked by each subject’s grey matter and applied to the PET data, in order to calculate standardized uptake value ratios (SUVRs) for each Schaefer atlas region.^42^ Aβ status was determined on global Aβ-PET SUVRs using cut-offs previously established in the ADNI cohort.^43,44^

### Tau epicenters

To identify tau epicenters for each TBI subgroup, we adopted a previously described method that is based on the assumption that brain regions exhibiting early abnormal tau development would manifest abnormal tau levels across a large proportion of the cohort, whereas regions with relatively late abnormal tau development would show abnormal tau levels in a relatively smaller subset of individuals.^19,20^ Within each TBI subgroup, for each individual separately, we rank ordered all Schaefer ROIs based on their tau-PET SUVRs, revealing the estimated cross-sectional tau spreading sequence. Subsequently, epicenters were defined as the top 10% of ROIs (i.e. *n* = 20 ROIs) exhibiting the highest tau-PET SUVRs, after which they were mapped on the group-level.

### Statistical analysis

Group differences in demographics were assessed using ANOVAs or two-sample *t*-tests for continuous variables, and Pearson’s χ^2^ tests of independence or Fisher’s exact tests for categorical variables. Group differences in global Aβ- and tau-PET signal were assessed using ANCOVAs adjusted for age, cognitive diagnosis, *APOE*ɛ4 carrier status, the time lag between the PET scan and cognitive diagnosis date and in the tau-PET model additionally for the global Aβ-PET level and the time lag between the Aβ- and tau-PET scan. Group differences in regional Aβ- and tau-PET signal were also assessed using ANCOVA and were adjusted for age, *APOE*ɛ4 carrier status and the global Aβ-PET or tau-PET level, respectively. The tau-PET model was additionally adjusted for the corresponding regional Aβ-PET SUVR and the time lag between the Aβ- and tau-PET scan. The raw mean differences in Aβ- and tau-PET signal between TBI subgroups were calculated by group-averaging the values within each of the 200 Schaefer ROIs and then subtracting them between groups. Moderating effects of TBI status on the association between regional Aβ-PET and tau-PET were tested using linear regression while adjusting for age, *APOE*ɛ4 carrier status, global tau-PET and the time lag between the Aβ- and tau-PET scan. Analyses that incorporated *APOE*ɛ4 carrier status in the model were conducted on a slightly smaller sample size due to missing data for five individuals (3 mild TBI, 2 moderate/severe TBI). Regional Aβ- and tau-PET analyses were FDR-corrected to account for multiple comparisons. Significance for all effects was determined at a two-tailed α=0.05. All statistical analyses were performed using R statistical software. Brain surface renderings were generated using the Connectome Workbench.

## Results

The sample included 65 individuals with a history of mild (*n* = 40; 38.8%) or moderate/severe (*n* = 25; 24.3%) TBI and 38 (36.9%) individuals without a history of TBI (see **Table 1** for demographics). About half of the individuals with a TBI history (33 individuals, 50.8%) experienced a TBI event while serving in Vietnam, while the others (32 individuals, 49.2%) were only exposed to TBI before or after Vietnam. Most individuals had experienced 1 (*n* = 35, 53.8%) or 2 (*n* = 15, 23.1%) TBI events, and the mean time difference between the last TBI event and the tau-PET scan was 39.63 ± 18.39 years. Mild cognitive impairment was more common in individuals with a history of moderate/severe TBI (10 individuals, 40.0%) compared with those without a TBI history (4 individuals, 10.5%), odds ratio (OR) = 5.50 [95% confidence interval (CI) 1.32 to 27.99], *P* = 0.01. Age at tau-PET, years of education, *APOE*ɛ4 carrier status, and race distribution did not differ across TBI groups (*P* > 0.05). The mean time difference between the Aβ-PET and tau-PET differed across groups [*F*(2, 100) = 14.18, *P* < 0.001]. Specifically, the mean time difference was longer in individuals without a history of TBI (2.34 ± 1.38 years) than in individuals with a history of mild TBI [0.94 ± 1.42 years; mean difference with no TBI = −1.40 (95% CI −2.13 to −0.67), *P* < 0.001] or moderate/severe TBI [0.76 ± 1.21 years; mean difference with no TBI = −1.58 (95% CI −2.41 to −0.75), *P* < 0.001]. An overview of relevant health factors across the TBI groups is presented in Supplementary Table 1.

**Table 1:** Demographics

	Total	No TBI	Mild TBI	Moderate/severe TBI	*P*
***N***	*103*	*38*	*40*	*25*	
**Age at tau-PET, years**	71.68 (4.95)	71.77 (5.34)	72.23 (5.01)	70.69 (4.20)	0.48^a^
**Male**	103 (100)	38 (100)	40 (100)	25 (100)	NA
**Education, years**	15.13 (2.45)	15.16 (2.35)	15.18 (2.66)	15.00 (2.33)	0.96^a^
**Race**					>0.05^b^
American Indian or Alaskan Native	2 (1.9)	1 (2.6)	0 (0.0)	1 (4.0)	
Black or African American	6 (5.8)	2 (5.3)	2 (5.0)	2 (8.0)	
More than one race	2 (1.9)	1 (2.6)	0 (0.0)	1 (4.0)	
White	92 (89.3)	33 (86.8)	38 (95.0)	21 (84.0)	
Unknown	1 (1.0)	1 (2.6)	0 (0.0)	0 (0.0)	
***APOE*ɛ4 prevalence**	23 (23.5)	6 (15.8)	9 (24.3)	8 (34.8)	0.23^c^
Missing	5 (4.9)	0 (0.0)	3 (7.5)	2 (8.0)	
**MCI**	23 (22.3)	4 (10.5)	9 (22.5)	10 (40.0)*	0.01^b**^
**TBI occurred in Vietnam**	33 (50.8)	NA	21 (52.5)	12 (48.0)	0.92^c^
**TBI frequency (at time of tau-PET)**		NA			0.77^b^
1	35 (53.8)		23 (57.5)	12 (48.0)	
2	15 (23.1)		8 (20.0)	7 (28.0)	
3	11 (16.9)		7 (17.5)	4 (16.0)	
4	1 (1.5)		0 (0.0)	1 (4.0)	
5	2 (3.1)		1 (2.5)	1 (4.0)	
7	1 (1.5)		1 (2.5)	0 (0.0)	
**Global Aβ-PET, SUVR**	1.12 (0.15)	1.09 (0.14)	1.12 (0.18)	1.17 (0.13)	0.16^a^
**Global tau-PET, SUVR**	1.08 (0.09)	1.07 (0.09)	1.07 (0.10)	1.10 (0.08)	0.38^a^
**Time lag Aβ- and tau-PET, years**	1.41 (1.52)	2.34 (1.38)	0.94 (1.42)*	0.76 (1.21)*	<0.001^a^
**Time lag last TBI and tau-PET, years**	39.63 (18.39)	NA	42.02 (18.04)	35.80 (18.65)	0.19^d^

Values are mean (standard deviation) for continuous variables and n (%) for categorical variables. Aβ, amyloid-β; ANOVA, analysis of variance; APOE, apolipoprotein E; MCI, mild cognitive impairment; NA, not applicable; PET, positron emission tomography; SUVR, standardized uptake value ratio; TBI, traumatic brain injury. ^a^Differences between groups were assessed using ANOVAs, ^b^Fisher’s exact tests, ^c^Pearson’s χ^2^ tests of independence, or ^d^two-sample *t*-test. *Significantly different from no TBI. **This *P*-value only applies to the comparison no TBI versus moderate/severe TBI; the other comparisons were not significant (*P* > 0.05).

### A history of TBI is not associated with overall increased Aβ or tau burden

**Figure 1:**
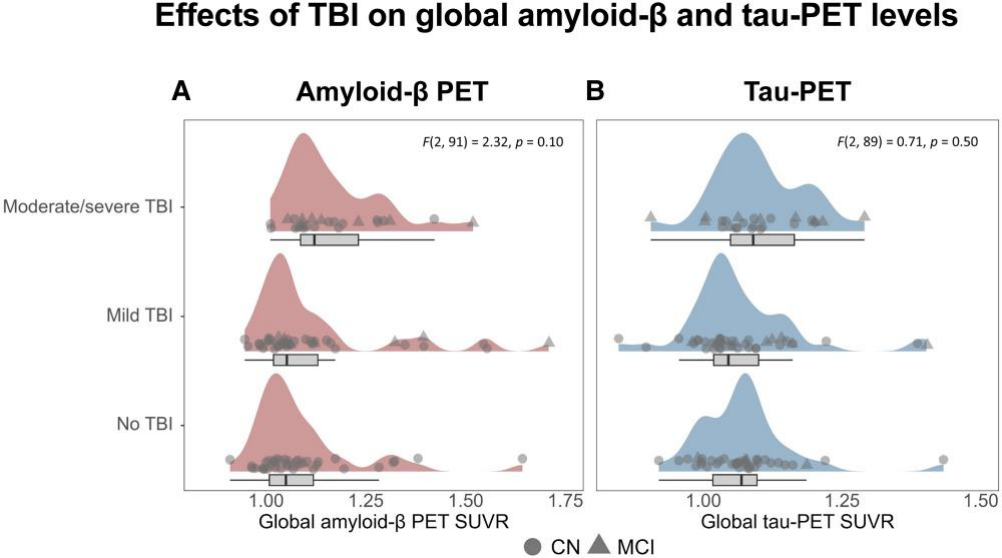
**Global Aβ-PET (A) and tau-PET (B) across TBI subgroups.** Differences in global Aβ-PET between groups were assessed by ANCOVAs adjusted for age, cognitive diagnosis, *APOE*ɛ4 carrier status, and the time lag between the Aβ-PET scan and cognitive diagnosis date. Differences in global tau-PET between groups were also assessed using ANCOVAs and adjusted for age, cognitive diagnosis, *APOE*ɛ4 carrier status, global Aβ-PET, the time lag between the Aβ- and tau-PET scan, and the time lag between the tau-PET scan and cognitive diagnosis date. The analyses were conducted on a sample of 98 individuals. Boxplots are displayed as median (centre line) ± interquartile range (box boundaries) with whiskers including observations falling within the 1.5 interquartile range. Data points marked with circles represent CN individuals, while data points marked with triangles represent individuals with MCI. Aβ, amyloid-β; ANCOVA, analysis of covariance; *APOE,* apolipoprotein E; CN, cognitively normal; MCI, mild cognitive impairment; PET, positron emission tomography; SUVR, standardized uptake value ratio; TBI, traumatic brain injury.

We first assessed whether a history of TBI was associated with elevated global or regional Aβ- or tau-PET signal. ANCOVAs demonstrated that the moderate/severe TBI group did have numerically higher Aβ-PET and tau-PET levels than the no TBI and mild TBI groups, but these differences were not statistically significant [Aβ-PET Fig. 1A, mean no TBI = 1.09 ± 0.14, mild TBI = 1.12 ± 0.18, moderate/severe TBI = 1.17 ± 0.13, *F*(2, 91) = 2.32, *P* = 0.10; tau-PET Fig. 1B, mean no TBI = 1.07 ± 0.09, mild TBI = 1.07 ± 0.10, moderate/severe TBI = 1.10 ± 0.08, *F*(2, 89) = 0.71, *P* = 0.50]. Congruently, we did not detect group differences in regional Aβ- and tau-PET SUVRs across 200 Schaefer ROIs when adjusting for multiple comparisons (FDR-corrected *P*-values > 0.05). Repeating these analyses while combining the mild- and moderate/severe TBI groups yielded similar results (Supplementary Fig. 1). The findings above substantiate the notion that TBI is not associated with significant increases in overall Aβ and tau load.

### TBI is associated with spatial heterogeneity in Aβ and tau-PET patterns

**Figure 2:**
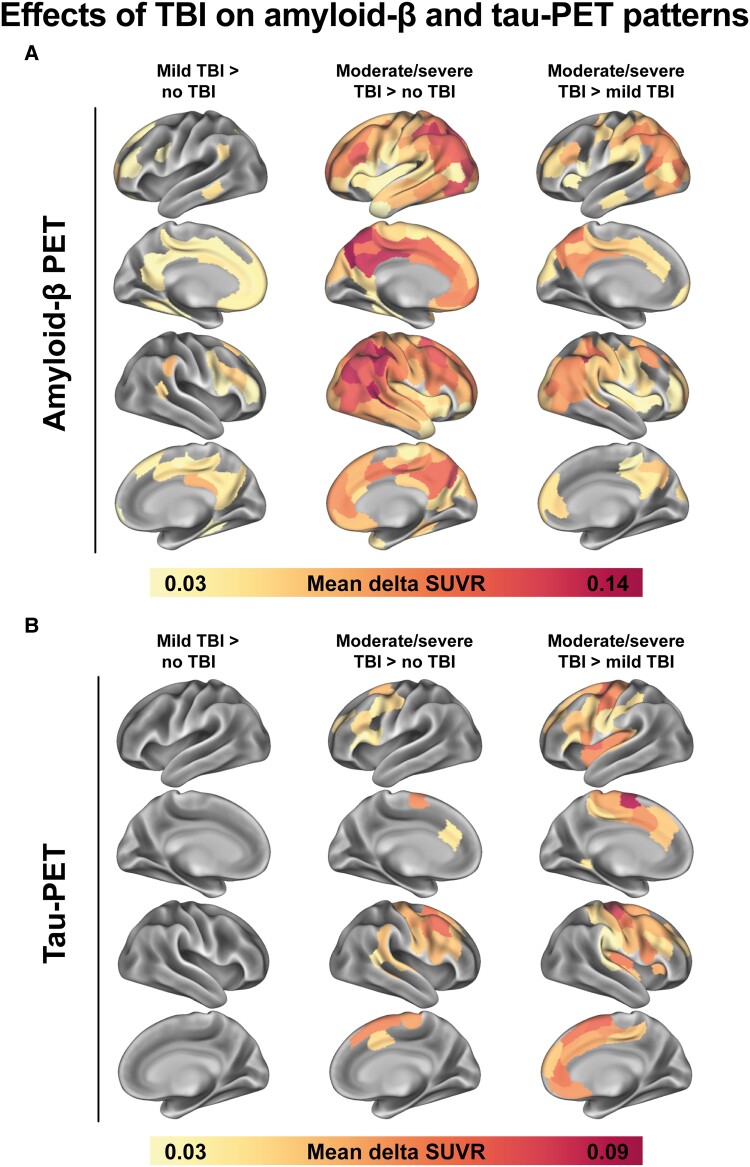
**Raw mean differences between TBI subgroups in Aβ-PET (A) and tau-PET (B) SUVRs within the 200 Schaefer atlas regions.** Differences between groups were assessed using ANCOVAs and were adjusted for age, *APOE*ɛ4 carrier status, and the global Aβ-PET (**A**) or tau-PET (**B**) level. The tau-PET model (**B**) was additionally adjusted for the corresponding regional Aβ-PET SUVR and the time lag between the Aβ- and tau-PET scan. The analyses were conducted on a sample of 98 individuals (no versus mild TBI: *n* = 75, no versus moderate/severe TBI: *n* = 61, mild versus moderate/severe TBI: *n* = 60). Mean differences are displayed for the whole sample (*n* = 103), and only for regions that showed a significant, FDR-corrected, group difference as identified by ANCOVA. Aβ, amyloid-β; ANCOVA, analysis of covariance; *APOE,* apolipoprotein E; FDR, false discovery rate; PET, positron emission tomography; SUVR, standardized uptake value ratio; TBI, traumatic brain injury.

Our second aim was to test whether a history of TBI—independent of global differences in Aβ- or tau-PET signal—was associated with spatially heterogeneous Aβ- or tau-PET patterns that deviate from the stereotypical Alzheimer’s disease pattern. This is plausible given the variation in the location of TBI damage that could give rise to local pathological brain changes like tau hyperphosphorylation.^35,36,38^ To test this, we ran ROI-wise ANCOVAs for Aβ- and tau-PET while controlling for global Aβ- and tau-PET SUVRs, respectively, in order to specifically test whether TBI was associated with regional in- or decreases in Aβ and tau load while accounting for the overall severity in Aβ and tau pathology. Supporting the view that TBI is associated with local alterations in Aβ and tau deposition, we detected regional differences in the distribution of Aβ- and tau-PET signal between groups, with TBI subgroups exhibiting regionally increased levels compared with the no TBI group. The raw mean differences for FDR-corrected group differences that were statistically significant [*F*-statistic intervals for Aβ-PET: no TBI versus mild TBI: *F*(1, 70) = 6.92–22.06, no TBI versus moderate/severe TBI: *F*(1, 56) = 4.39–50.55, mild TBI versus moderate/severe TBI: *F*(1, 55) = 5.66–29.01; *F*-statistic intervals for tau-PET: no TBI versus mild TBI: all non-significant, no TBI versus moderate/severe TBI: *F*(1, 54) = 7.71–30.65, mild TBI versus moderate/severe TBI: *F*(1, 53) = 6.11–35.78] are shown in Fig. 2. We appreciated a stepwise increase in frontal and parietal Aβ-PET signal from no TBI to mild TBI to moderate/severe TBI at a given overall level of Aβ pathology. For tau-PET, the moderate/severe TBI group showed higher regional signal in frontal areas than the no TBI and mild TBI groups. Moreover, Supplementary Fig. 2 shows the average Aβ-PET and tau-PET SUVR among the significant ROIs (FDR-corrected *P* < 0.05) from the ANCOVAs across TBI groups. While repeating these analyses comparing the whole TBI group with the no TBI group, regional differences in Aβ-PET signal were weaker and there were no differences in regional tau-PET signal (Supplementary Fig. 3). Together, these findings suggest that even though TBI is not associated with significant elevated Aβ or tau levels, it is related to altered spatial patterns of Aβ and tau deposition.

### Tau-PET epicenters are heterogeneous in TBI

**Figure 3:**
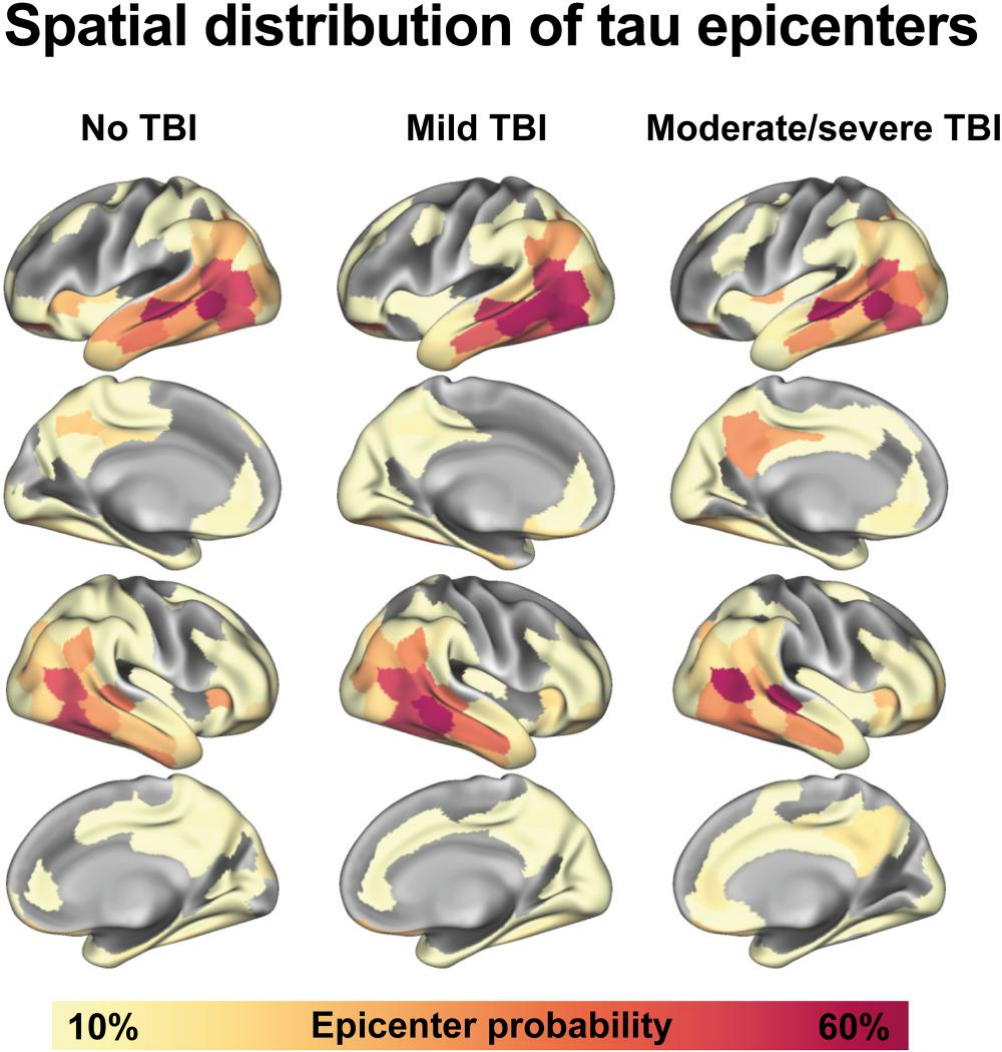
**Tau epicenters for all TBI subgroups.** Tau-PET SUVRs were assessed within the 200 Schaefer atlas regions. Group-wise epicenter regions (i.e. regions with the 10% highest tau-PET SUVRs) are shown, accompanied by their respective epicenter probabilities. Data are displayed for the whole sample (*n* = 103). PET, positron emission tomography; SUVR, standardized uptake value ratio; TBI, traumatic brain injury.

Due to emerging evidence suggesting that tau propagates through functionally connected regions starting from subject-specific epicenters—i.e. the brain regions containing the earliest, highest and most consistent tau-PET signal—our third aim was to investigate the spatial heterogeneity in the putative sites of tau onset in TBI by assessing tau epicenters across the three TBI subgroups. As expected, we found that regions within the temporal lobe consistently exhibited the highest epicenter probability in all subgroups, with the temporal lobe patterns being slightly more pronounced in the no TBI and mild TBI group compared with the moderate/severe TBI group (Fig. 3). Interestingly, when visually compared with the no TBI and mild TBI groups, the moderate/severe TBI group showed somewhat more involvement of the posterior cingulate and precuneus. These are Braak stage V (out of VI) regions in the stereotypical tau staging system.^11^ Repeating the analyses while merging the mild TBI and moderate/severe groups showed relatively high resemblance between the groups with and without TBI, lacking the presence of posterior cingulate and precuneus epicenters (Supplementary Fig. 4). These findings suggest that there might be some variability in the initiation site of earliest tau pathology, and thus in subsequent tau spreading patterns, in TBI.

### TBI is associated with altered Aβ-tau relationships

**Figure 4:**
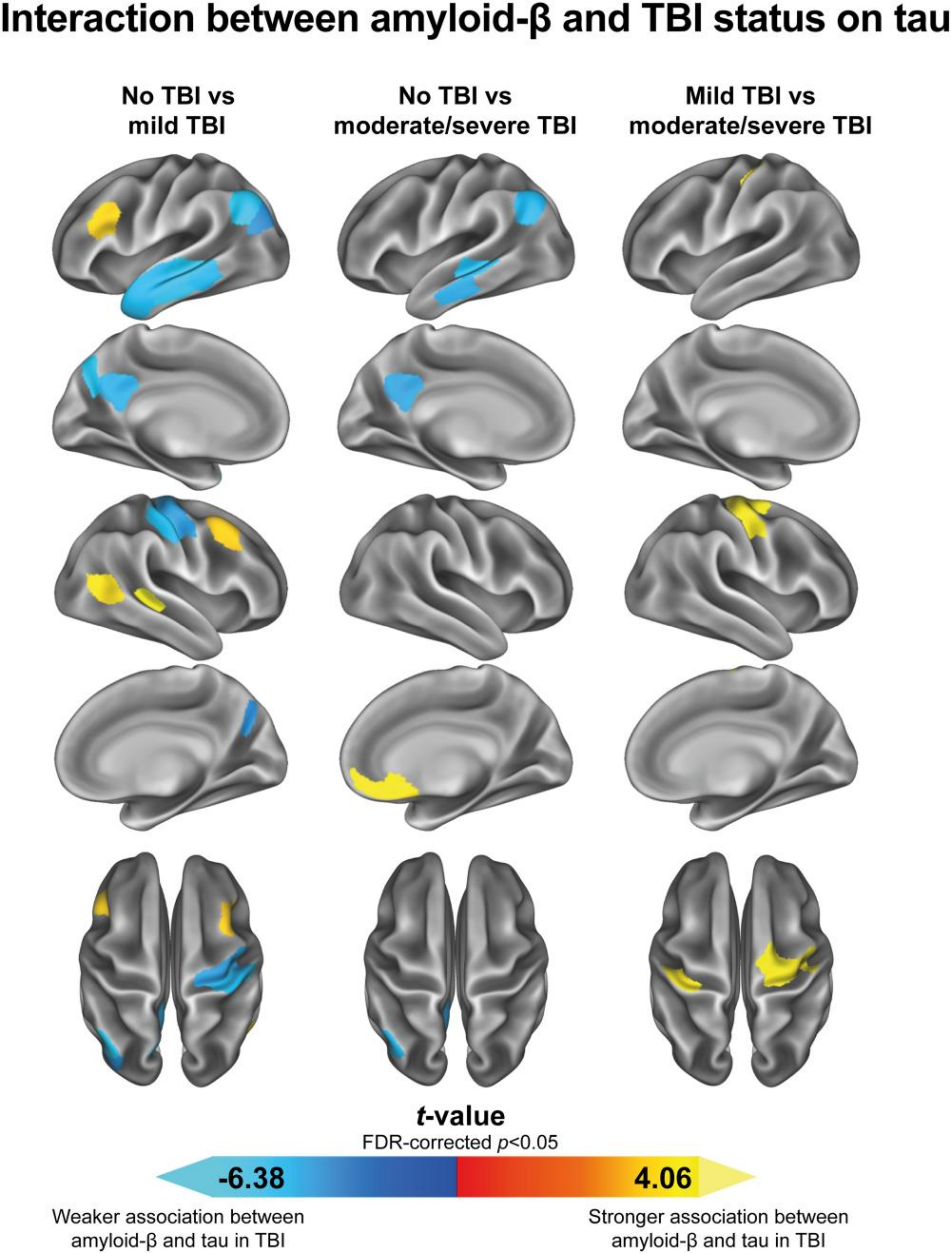
**Interaction between Aβ-PET and TBI status on tau-PET within the 200 Schaefer atlas regions.** Aβ- and tau-PET SUVRs were assessed within the 200 Schaefer atlas regions. Interaction effects were examined in linear regression models while adjusting for age, *APOE*ɛ4 carrier status, global tau-PET, and the time lag between the Aβ- and tau-PET scan. The analyses were conducted on a sample of 98 individuals (no versus mild TBI: *n* = 75, no versus moderate/severe TBI: *n* = 61, mild versus moderate/severe TBI: *n* = 60). Only FDR-corrected significant group differences are shown. Positive *t*-values indicate stronger associations between Aβ-PET and tau-PET in the presence of a history of (more severe) TBI, while negative *t*-values indicate the opposite. Aβ, amyloid-β; *APOE,* apolipoprotein E; FDR, false discovery rate; PET, positron emission tomography; SUVR, standardized uptake value ratio; TBI, traumatic brain injury.

There is a well-established connection between Aβ and hyperphosphorylated tau in the context of cognitive aging and Alzheimer’s disease.^11^ Notably, it is widely understood that Aβ drives the accumulation of tau in the neocortex, with both protein aggregates demonstrating a distinct spreading pattern.^11,22,23^ Therefore, a logical next step was to assess whether a history of TBI might influence the relationship between Aβ and tau. This is particularly interesting considering the observed spatial heterogeneity of Aβ- and tau-PET signal in our TBI individuals. Thus, our fourth aim was to assess the moderating effects of TBI status on the association between Aβ- and tau-PET. Using linear regression analyses, we found that, when adjusting for global tau-PET, a history of either mild or moderate/severe TBI was associated with a weakening of the relationship between Aβ-PET and tau-PET in early Alzheimer’s disease–susceptible temporo-parietal brain regions (Fig. 4). In contrast, a stronger association between Aβ-PET and tau-PET was observed in dorsolateral prefrontal and orbitofrontal regions, as well as in the temporo-parietal junction, in mild TBI and moderate/severe TBI compared with no TBI. Furthermore, the moderate/severe TBI group presented with a stronger association between Aβ-PET and tau-PET in parts of the sensorimotor cortex when compared with the mild TBI group. Supplementary Fig. 5 shows the interaction between average Aβ-PET and TBI on average tau-PET among significant ROIs (FDR-corrected *P* < 0.05) from the linear regression analyses. Comparable results were found when repeating these same analyses examining the no TBI group versus the whole TBI group, instead of the two TBI groups separately (Supplementary Fig. 6). Taken together, we found that the relationship between Aβ-PET and tau-PET was different in individuals with a history of TBI compared with individuals without a history of TBI. More specifically, the relationship between Aβ- and tau-PET tended to be weaker in Alzheimer’s disease–typical temporo-parietal brain regions, while it was stronger in brain regions that typically become abnormal in late(r) stages of Alzheimer’s disease.

## Discussion

The primary goal of the current study was to investigate the association between TBI in early/mid-life (mean time lag between last TBI and tau-PET scan of 39.63 ± 18.39 years) and global and regional Aβ- and tau-PET signal in aging, as well as the moderating effects of TBI on the relationship between Aβ- and tau-PET. Our main findings were that a history of TBI was associated with spatial heterogeneity in Aβ- and tau-PET signal (i.e. TBI groups had more relative frontal and parietal signal than the no TBI group) but not with overall increased Aβ- and tau-PET load. Moreover, the relationship between Aβ- and tau-PET differed between individuals with and without a history of TBI, where subjects with a history of TBI had stronger Aβ-related tau accumulation in regions that typically accumulate tau late in Alzheimer’s disease, while the relationship between Aβ and tau was weaker in temporo-parietal brain regions that accumulate tau early in Alzheimer’s disease. Taken together, given the multifaceted influence of TBI on brain health, the heterogeneity in Aβ- and tau-PET signal in the context of the highly variable nature of TBI could indicate that spreading patterns may be influenced by the location of TBI-related brain damage and the functional connections of the tau epicenters. Specifically, TBI may cause local injury to the brain, leading to different pathology spreading patterns. This suggests that external influences such as head impacts may actually influence Alzheimer’s disease pathophysiology.

The most striking finding of this study was the observed moderating effects of TBI status on the relationship between Aβ- and tau-PET. Specifically, a history of TBI was associated with a weaker relationship between Aβ- and tau-PET in temporo-parietal brain regions and with a stronger relationship between Aβ- and tau-PET in dorsolateral prefrontal and orbitofrontal regions, and in the temporo-parietal junction. It has been validated in post-mortem studies that in typical amnestic Alzheimer’s disease, Aβ and tau follow a distinct topographical spreading pattern throughout the brain from the preclinical to the dementia stage.^11^ Particularly, Aβ starts in the neocortex and progresses from there to the allocortex and subcortical regions, and lastly to the cerebellum and brainstem.^11^ In contrast, tau pathology generally starts in the (trans)entorhinal cortex (Braak stage I-II).^11^ It is hypothesized that when Aβ is widely present across the neocortex, it facilitates the spread of tau outside of the medial temporal lobe to limbic regions (Braak stage III-IV) and lastly to the neocortex including sensorimotor regions (Braak stage V-VI).^11,22,23^ Previous research has shown that Aβ- and tau-PET are positively correlated throughout the whole cortex, but specific spatial associations differ across disease stages and brain regions.^34^ Associations between Aβ and tau have been found to be the strongest in temporo-parietal brain regions in early disease stages.^34,45,46^ Therefore, it is quite remarkable that we found the reverse in TBI, whereby Aβ and tau in earlier Braak regions exhibited a decoupling, and a tighter connection between the two proteins was observed in the later Braak regions—which is particularly notable given that global tau levels were low in all groups. A suitable explanation is that TBI is associated with heterogeneous Aβ and tau spreading patterns. Specifically, the relationship between Aβ and tau depends on their respective locations in the brain^34^ and thus heterogeneous spreading patterns of Aβ and tau could give rise to differential Aβ-tau interactions.

In fact, in line with this, when assessing differences between TBI groups in regional Aβ- and tau-PET values while adjusting for global Aβ- and tau-PET levels, respectively, we found that individuals with a history of TBI had more frontal and parietal Aβ- and tau-PET signal compared with individuals without a history of TBI. Additionally, our epicenter analyses showed that even though for all groups the majority of regions with the highest tau-PET signal were situated in the temporal lobe—usually the first location for tau deposition in aging^16,21^—the moderate/severe TBI group showed most spatial variability in epicenter regions. Specifically, the temporal pattern was less pronounced in moderate/severe TBI compared with the no- and mild TBI groups, and the posterior cingulate and precuneus (Braak stage V) clearly appeared as epicenters as well. This indicates that at least for a subset of individuals with moderate/severe TBI, the highest and thus the assumed earliest tau-PET signal was located in these relatively late stage regions, which deviates from the stereotypical pathological staging system of tau pathology.^11^ Interestingly, this finding is in accordance with previous studies in TBI, showing most consistent evidence for increased Aβ in the cingulate gyrus, (pre)cuneus and the rest of the parietal lobe, and for increased tau throughout the whole brain but most consistently in the frontal and parietal lobes and the precuneus.^13^ Taking into account the substantial inter-individual heterogeneity in tau spreading patterns mirroring clinical phenotypes, as indicated in prior research (e.g. in atypical variants of Alzheimer’s disease),^19,24-31^ deviations from this stereotypical staging scheme are within the scope of expectation. Furthermore, brain damage following TBI can be highly heterogeneous, consisting of both focal and diffuse axonal injuries.^38^ Previous studies in which mice were exposed to TBI showed that Aβ and tau accumulated in close proximity to the region of injury.^35-37^ Interestingly, one of these studies showed that p-tau spread from the injury site to synaptically connected brain regions,^35^ congruent with the recent hypothesis that tau spreads through communicating neurons in humans.^19,20^ Therefore, it is plausible that in TBI, tau seeds close to the area of injury, after which it spreads transneuronally to accumulate predominantly in connected regions. The posterior cingulate and precuneus being highly connected and metabolically active brain regions^47^ lends further credence to this theory.

Although we saw a stepwise numerical increase in Aβ- and tau-PET from no TBI to mild TBI to moderate/severe TBI, differences between groups were not statistically significant. The lack of an observed impact of TBI on Alzheimer’s disease pathology aligns with findings from a previous study using ADNI DOD data.^48^ In that study, no significant increases were detected in Aβ ([^18^F]florbetaben, Centiloid; global neocortex), tau ([^18^F]flortaucipir, SUVR; global neocortex as well as three composites, i.e. mesial-temporal, temporo-parietal, and rest of the neocortex) or glucose metabolism ([^18^F]FDG, SUVR; global neocortex, frontal composite, mesial-temporal, posterior cortical regions). In contrast, some other studies did find elevated Aβ and tau associated with TBI.^49-61^ However, a closer examination of these previous studies reveals that these effects were predominantly observed in individuals with cognitive impairment,^49,51,54,56,57,62^ with positive Aβ biomarkers,^51,62^ or in homozygous *APOE*ɛ4 carriers.^54^ Specifically, these earlier findings suggest that TBI might accelerate Alzheimer’s disease–related processes particularly in individuals already predisposed or vulnerable to Alzheimer’s disease. In the current study, the majority of individuals (~78%) were cognitively normal, and we did not preselect based on Aβ status. Additionally, in line with population estimates,^63^ 23.5% of individuals were *APOE*ɛ4 carriers (either homozygous or heterozygous). These aspects of our study population could have contributed to our results deviating from previous findings.

A strength of the current study was the focus on investigating the association between TBI and spatial variability in Aβ- and tau-PET signal as well as Aβ-tau interactions, thereby addressing an important gap in knowledge. However, there are also several limitations to consider. First, there were some constraints with regard to the TBI classification. TBI history was assessed by telephonic interviews with participants, introducing the risk of recall bias. Furthermore, the type of TBI was not specified in the available data, while each type of TBI could give rise to different types of brain damage. In addition, we classified TBI severity using the criteria from the Department of Defense and Veterans Affairs, which is one of several guidelines available for head injury classification. Also, not all details from these criteria were available in the ADNI DOD dataset [for example, the criteria distinguish between amnesia >1 day (moderate) and >7 days (severe), but the maximal category we had access to was >1 day]. Therefore, there is a possibility that we have been unable to characterize the most severe TBI cases, which could have been relevant considering the stepwise increase in Aβ- and tau-PET signal that we found. Moreover, we cannot rule out the risk that our inclusion criteria encompassed very mild TBIs that may not be of significant relevance in the context of TBI. When mixed with other TBIs that are of interest, these mild cases might have diluted or averaged out potential effects. A second limitation is that the time lag between the Aβ-PET and tau-PET scan was significantly longer in the no TBI compared with the mild TBI and moderate/severe TBI group. However, we accounted for this observation by including the time lag between the Aβ-PET and tau-PET scan as a covariate into our statistical models. Furthermore, a key limitation of this study is the sample size. While our total cohort of 103 participants is considerable given the stringent inclusion criteria requiring each participant to have Aβ-PET, tau-PET, as well as TBI history data available, we recognize that the TBI subgroups are relatively small. Unfortunately, the limited availability of Aβ-PET and especially tau-PET in this population restricted our ability to include a larger sample in this study. To mitigate this, we grouped the mild TBI and moderate/severe TBI participants together in additional analyses, which overall confirmed our subgroup results. Also, we made sure to implement stringent *P*-value corrections—including the Benjamini–Hochberg FDR correction—to minimize the risk of false positives and strengthen the robustness of our results. Thus, while it is crucial that our work will be replicated in larger cohorts, our findings provide a valuable foundation for subsequent research, which could potentially be made feasible through multicentre collaborations. Another constraint is the study’s cross-sectional design, which prevents the assessment of changes in the relationship between TBI and Alzheimer’s disease pathology over time. Longitudinal PET data, ideally combined with functional MRI, are needed to make more accurate inferences about intra-individual Aβ and tau accumulation and their associations with TBI. An additional limitation relates to the exclusion of women in this study (i.e. *n* = 2). It is well-documented that men are more likely to be veterans and experience TBI at a higher rate.^64^ However, when women do sustain TBI, they tend to be more susceptible to negative effects on health.^64,65^ Also, Alzheimer’s disease is more prevalent in women, influenced by factors such as longevity, selective survival of men with healthier cardiovascular risk profiles, genetic factors (e.g. *APOE*ɛ4 posing a higher risk in women), and hormonal changes like decreased oestrogen post-menopause.^66^ Given these distinctions, we recognize that the absence of women in our study represents a limitation. It is possible that the relationship between TBI and Aβ and tau pathology differs between sexes, and that TBI’s impact on Alzheimer’s disease pathology may be more pronounced in women. Future research should aim to address these important questions. Lastly, it is important to mention the possibility that the observed tau-PET signal may partially reflect non-tau related processes (e.g. astrogliosis, iron accumulation, spill-in from off-target regions), given the suboptimal binding properties of [^18^F]flortaucipir to non-Alzheimer’s disease tau pathology.^67,68^

To conclude, our study did not reveal a notable association between TBI and globally increased Aβ- and tau-PET signal. However, while acknowledging the relatively small sample size and generally low levels of Alzheimer’s disease pathology in this sample, our data suggest that Aβ and tau pathology may be spatially heterogeneous in individuals that experienced TBI, deviating from the typical deposition pattern observed in amnestic Alzheimer’s disease. Moreover, we showed that TBI might be associated with a decoupling between established Aβ and tau relationships in early Alzheimer’s disease–susceptible brain regions, and with a tighter link between the two proteins in brain regions less specific to Alzheimer’s disease but more commonly linked to TBI.^13^ Future studies should seek to validate and replicate these findings in larger cohorts using a longitudinal design, ideally encompassing both male and female participants. In addition, these studies should aim to include detailed information on the type and frequency of TBI, and incorporate a broader range of more severe TBIs.

## Supplementary Material

fcaf009_Supplementary_Data

## Data Availability

Data used in this article were obtained from the ADNI database (adni.loni.usc.edu). The ADNI was launched in 2003 as a public-private partnership, led by Principal Investigator Michael W. Weiner, MD. The goal of ADNI has been to test whether serial MRI, PET, other biological markers, and clinical and neuropsychological assessment can be combined to measure the progression of mild cognitive impairment and early Alzheimer’s disease. ADNI(-DOD) data are publicly available. Code generated for this work can be found at: https://github.com/OssenKoppeLab/HdeBruin_ADNI_DOD.
