# Supplementary material for: Amyloid-β and tau deposition in traumatic brain injury: a study of Vietnam War veterans
Source: Brain Commun. 2025 Jan 10;7(1):fcaf009. doi: 10.1093/braincomms/fcaf009 (PMC11752645; doi:10.1093/braincomms/fcaf009)
Supplement: fcaf009_Supplementary_Data [file fcaf009_supplementary_data.docx]

|  | **Total** | **No TBI** | **Mild TBI** | **Moderate/severe TBI** | ***p*** |
| --- | --- | --- | --- | --- | --- |
| ***N*** | 103 | 38 | 40 | 25 |  |
| **Neurological medical history (other than AD)** | 42 (40.8) | 8 (21.1) | 19 (47.5) | 15 (60.0)* | <0.01^a^ |
| **Cardiovascular medical history** | 79 (76.7) | 31 (81.6) | 29 (72.5) | 19 (76.0) | 0.64^a^ |
| **Psychiatric medical history** | 61 (59.2) | 20 (52.6) | 28 (70.0) | 13 (52.0) | 0.21^a^ |
| **Current PTSD** | 47 (47.0) | 18 (48.6) | 21 (53.8) | 8 (33.3) | 0.28^a^ |
| Missing | 3 (2.9) | 1 (1.0) | 1 (1.0) | 1 (1.0) |  |
| **Current MDD** | 20 (19.6) | 8 (21.6) | 9 (22.5) | 3 (12.0) | 0.57^b^ |
| Missing | 1 (1.0) | 1 (1.0) | 0 (0.0) | 0 (0.0) |  |
| **Alcohol status** |  |  |  |  | 0.72^b^ |
| No alcohol abuse/dependence | 60 (58.3) | 23 (60.5) | 22 (55.0) | 15 (60.0) |  |
| Alcohol abuse | 17 (16.5) | 4 (10.5) | 9 (22.5) | 4 (16.0) |  |
| Alcohol dependence | 26 (25.2) | 11 (28.9) | 9 (22.5) | 6 (24.0) |  |

**Supplementary Table 1: Health factors across TBI groups.** Values are *n* (%). Differences between groups were assessed using Pearson’s Chi-squared tests of independence,^a^ and Fisher’s exact tests.^b^ *Significantly different from no TBI (post-hoc Fisher’s exact test with Bonferroni correction). Current PTSD was defined by meeting the criteria of the DSM-IV-TR and a CAPS score of greater than or equal to 40. MDD and alcohol status were determined using the SCID-IV. AD = Alzheimer’s disease. CAPS = Clinician Administered PTSD Scale. DSM-IV-TR = diagnostic and statistical manual of mental disorders, fourth edition, text revision. MDD = major depressive disorder. PTSD = post-traumatic stress disorder. SCID-IV = structured clinical interview for the DSM-IV. TBI = traumatic brain injury.


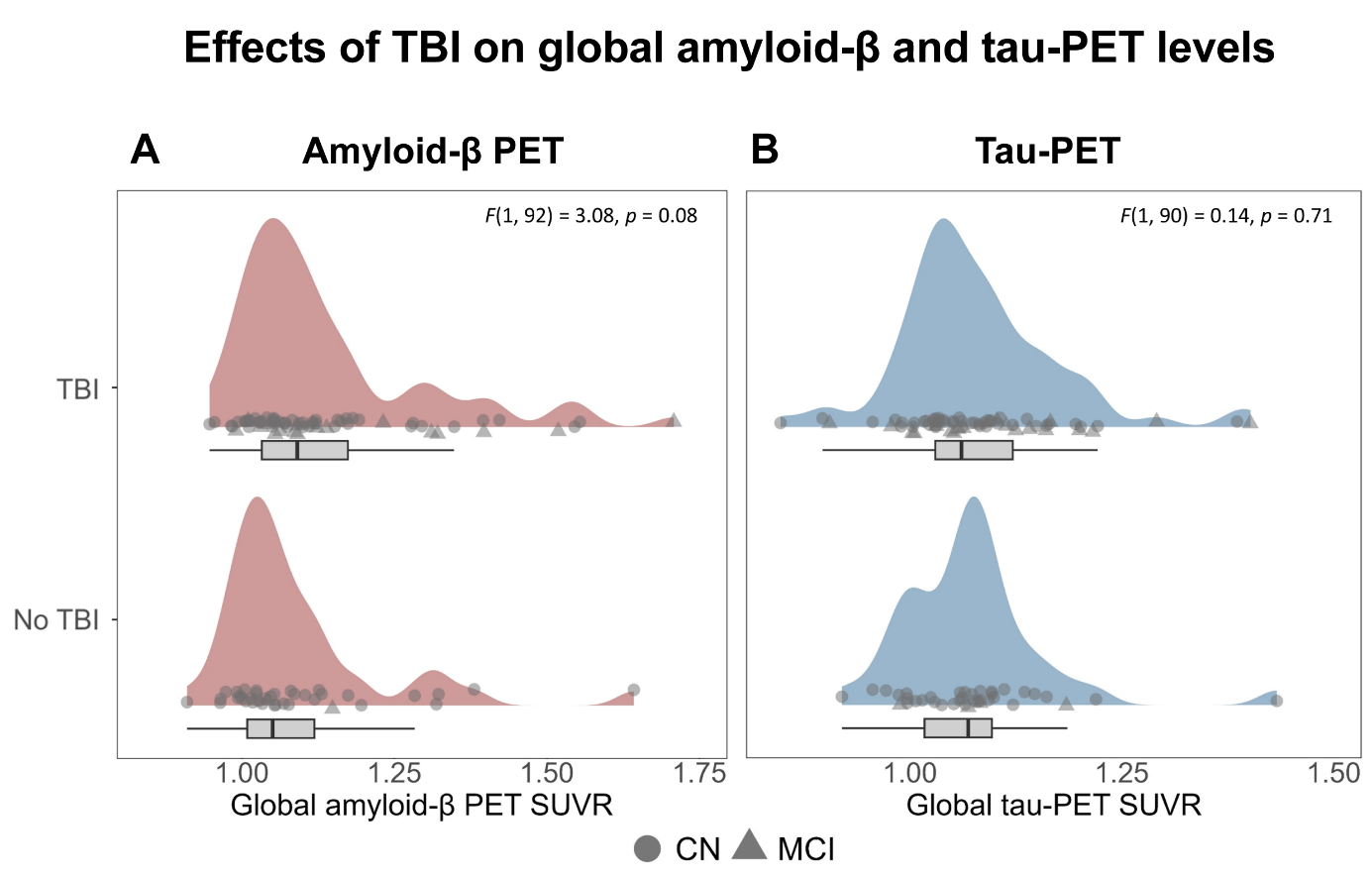
 **Supplementary Fig. 1: Global Aβ-PET (A) and tau-PET (B) across TBI subgroups.** Differences in global Aβ-PET between groups were assessed by ANCOVAs adjusted for age, cognitive diagnosis, *APOE*ε4 carrier status, and the time lag between the Aβ-PET scan and cognitive diagnosis date. Differences in global tau-PET between groups were also assessed using ANCOVAs and adjusted for age, cognitive diagnosis, *APOE*ε4 carrier status, global Aβ-PET, the time lag between the Aβ- and tau-PET scan, and the time lag between the tau-PET scan and cognitive diagnosis date. The analyses were conducted on a sample of 98 individuals. Boxplots are displayed as median (center line) ± interquartile range (box boundaries) with whiskers including observations falling within the 1.5 interquartile range. Data points marked with circles represent CN individuals, while data points marked with triangles represent individuals with MCI. Aβ = amyloid-β. ANCOVA = analysis of covariance. *APOE* = apolipoprotein E. CN = cognitively normal. MCI = mild cognitive impairment. PET = positron emission tomography. SUVR = standardized uptake value ratio. TBI = traumatic brain injury.

**
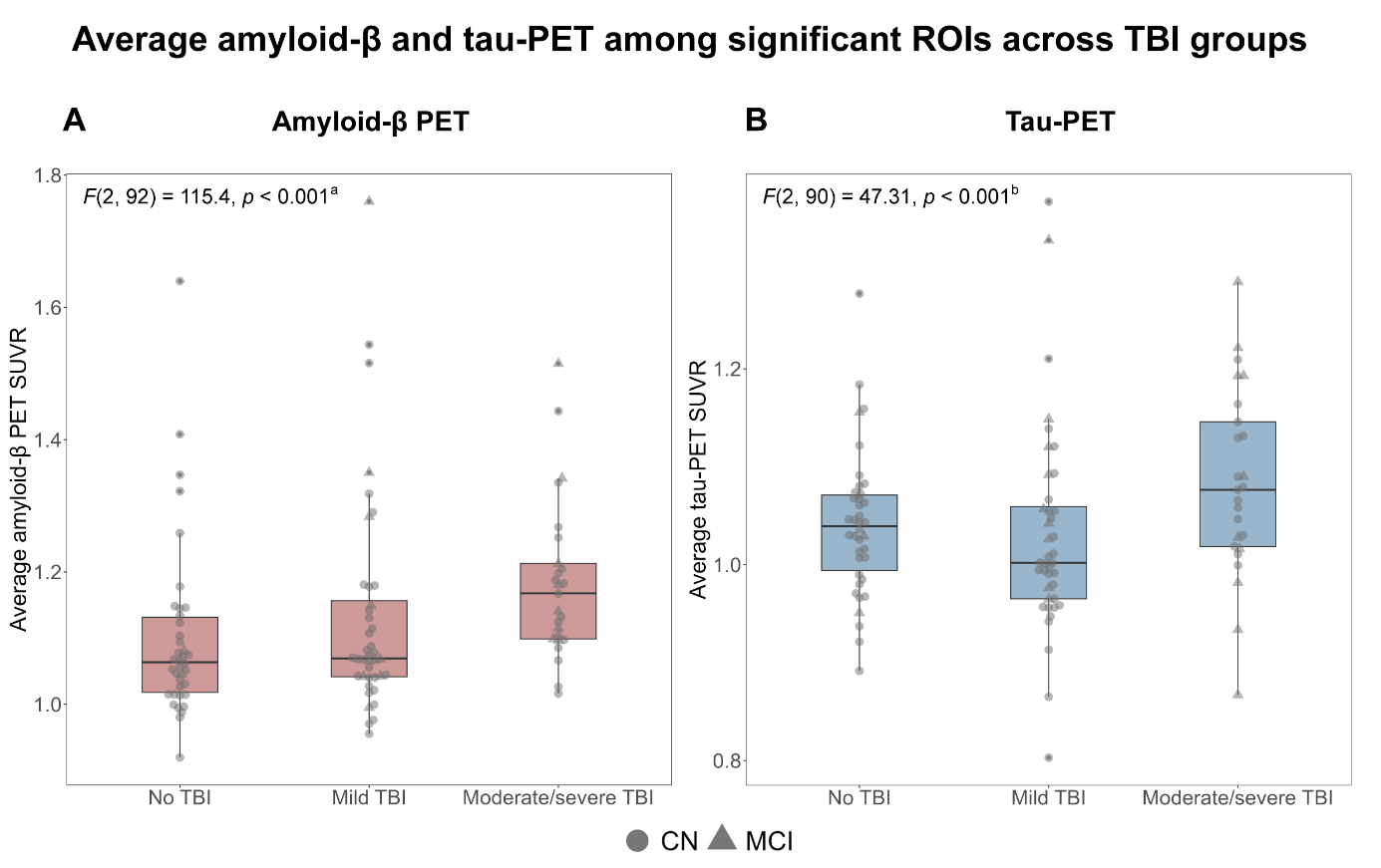
**

**Supplementary Fig. 2: Average Aβ-PET (A) and tau-PET (B) among significant ROIs across TBI groups.** ANCOVA was used to assess differences between TBI groups in Aβ-PET and tau-PET SUVRs within the 200 Schaefer atlas regions while adjusting for age, *APOE*ε4 carrier status, and the global Aβ-PET or tau-PET level, respectively. The tau-PET model was additionally adjusted for the corresponding regional Aβ-PET SUVR and the time lag between the Aβ- and tau-PET scan. Subsequently, we identified ROIs with significant differences between TBI groups (FDR-corrected *p* < 0.05). For both Aβ-PET and tau-PET, we averaged the SUVRs of these significant ROIs and then assessed the differences in average Aβ-PET (A) and tau-PET (B) SUVRs between TBI groups. We used the same covariates as mentioned earlier, except that we now adjusted for the global instead of the regional Aβ-PET SUVR in the tau-PET model. The analyses were conducted on a sample of 98 individuals. Boxplots are displayed as median (center line) ± interquartile range (box boundaries) with whiskers including observations falling within the 1.5 interquartile range. Data points marked with circles represent CN individuals, while data points marked with triangles represent individuals with MCI. ^a^All group differences were statistically significant. ^b^Moderate/severe TBI was significantly different from no TBI and mild TBI. Aβ = amyloid-β. ANCOVA = analysis of covariance. *APOE* = apolipoprotein E. CN = cognitively normal. FDR = false discovery rate. MCI = mild cognitive impairment. PET = positron emission tomography. ROI = region of interest. SUVR = standardized uptake value ratio. TBI = traumatic brain injury.


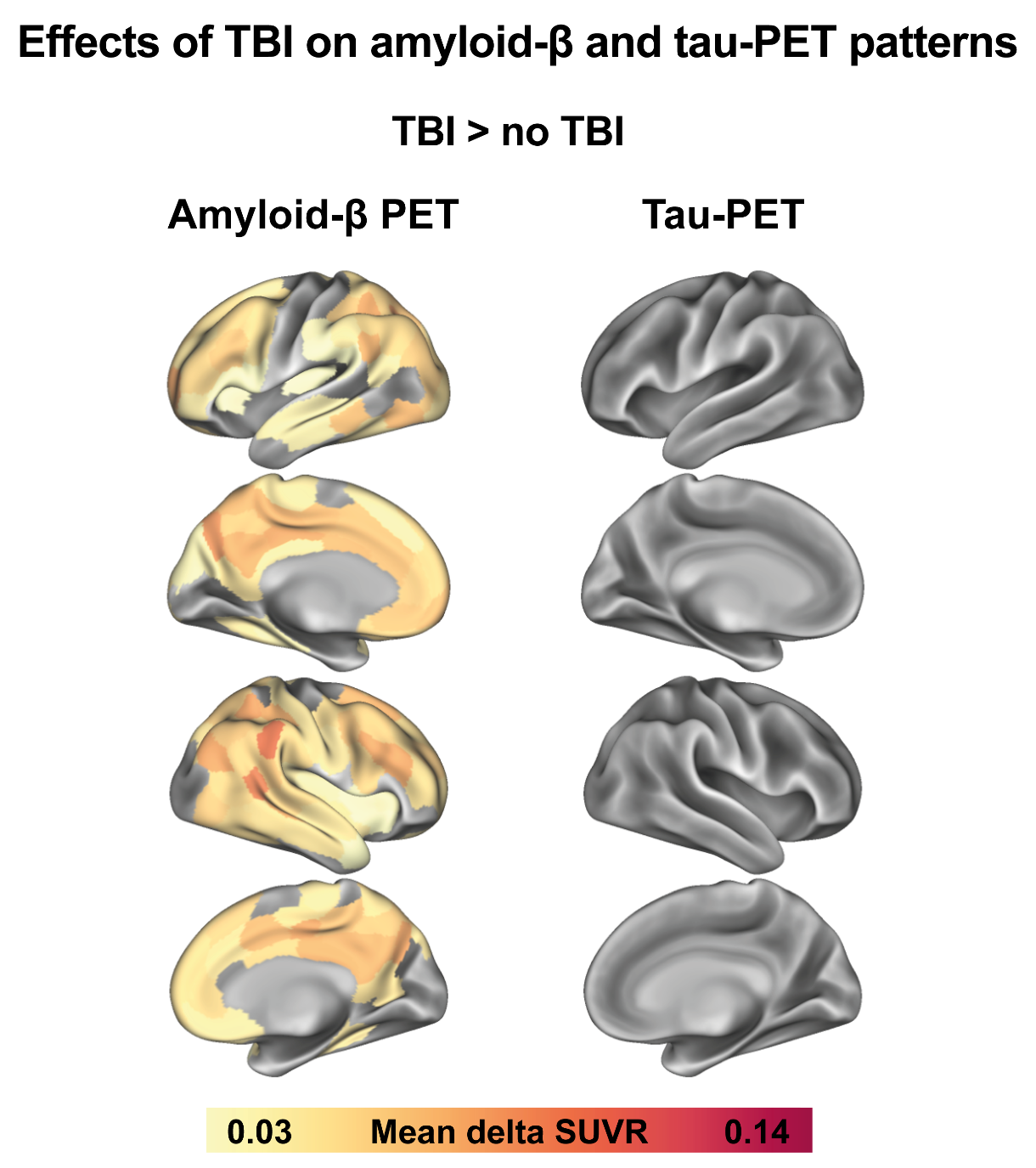


**Supplementary Fig. 3: Raw mean differences between TBI subgroups in Aβ-PET and tau-PET SUVRs within the 200 Schaefer atlas regions.** Differences between groups were assessed using ANCOVAs and were adjusted for age, *APOE*ε4 carrier status, and the global Aβ-PET (Aβ-PET model) or tau-PET (tau-PET model) level. The tau-PET model was additionally adjusted for the corresponding regional Aβ-PET SUVR and the time lag between the Aβ- and tau-PET scan. The analyses were conducted on a sample of 98 individuals. Mean differences are displayed for the whole sample (*n* = 103), and only for regions that showed a significant, FDR-corrected, group difference as identified by ANCOVA. Only Aβ-PET showed significant differences between groups. Aβ = amyloid-β. ANCOVA = analysis of covariance. *APOE* = apolipoprotein E. FDR = false discovery rate. PET = positron emission tomography. SUVR = standardized uptake value ratio. TBI = traumatic brain injury.


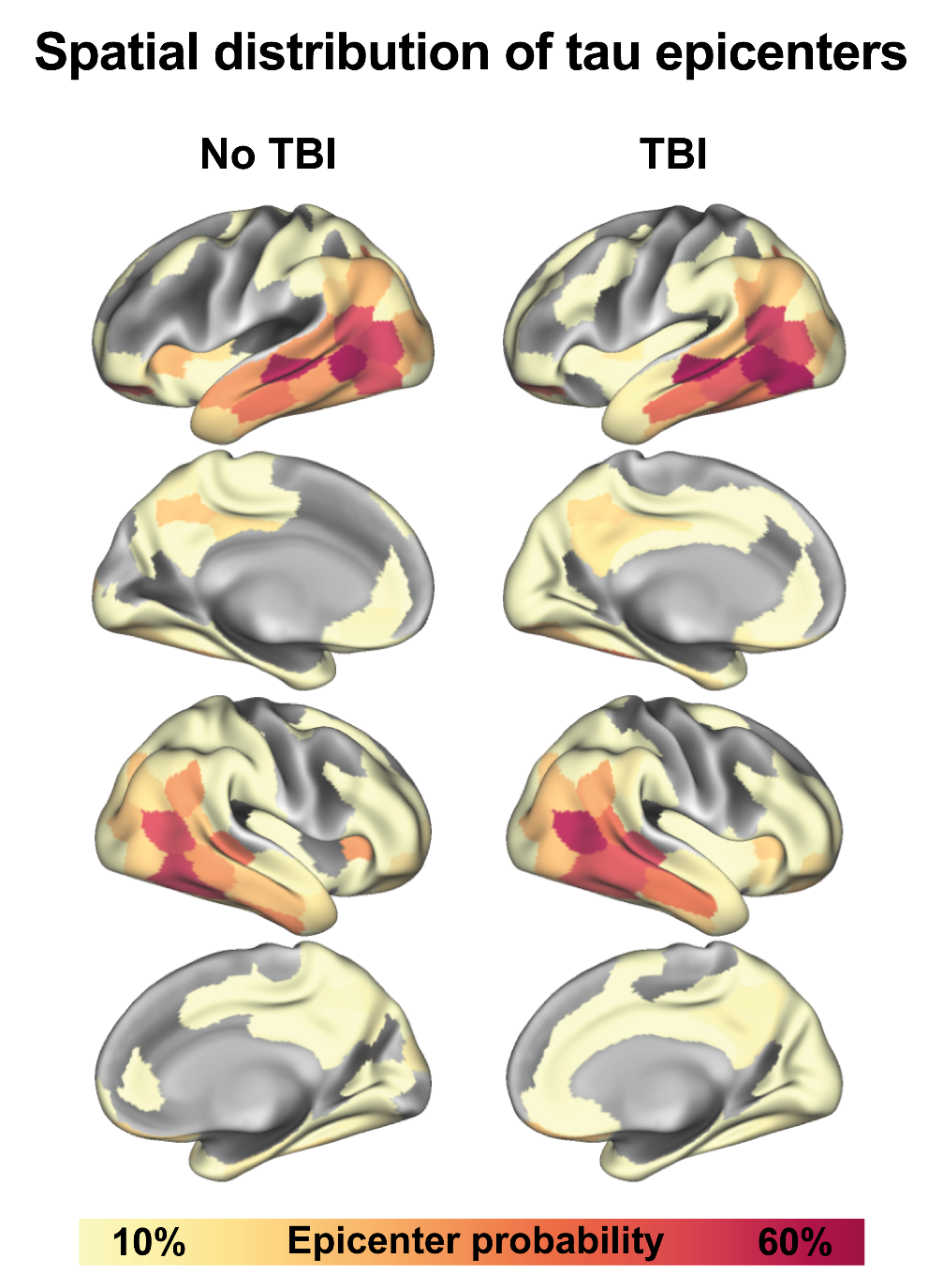


**Supplementary Fig. 4: Tau epicenters for TBI subgroups.** Tau-PET SUVRs were assessed within the 200 Schaefer atlas regions. Group-wise epicenter regions (i.e. regions with the 10% highest tau-PET SUVRs) are shown, accompanied by their respective epicenter probabilities. Data are displayed for the whole sample (*n* = 103). PET = positron emission tomography. SUVR = standardized uptake value ratio. TBI = traumatic brain injury.


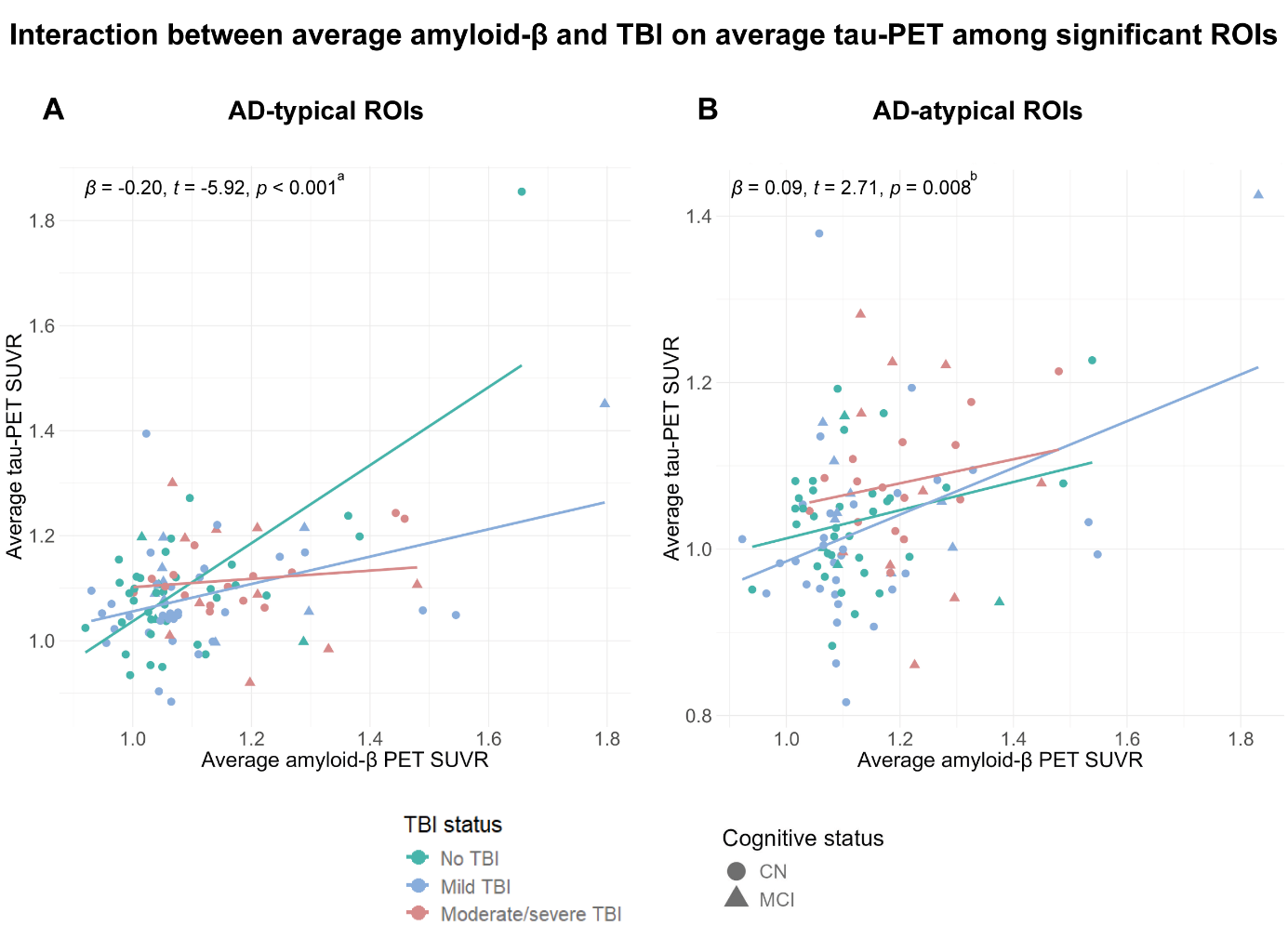


**Supplementary Fig. 5: Interaction between average Aβ-PET SUVR and TBI status on average tau-PET SUVR among significant ROIs.** Aβ- and tau-PET SUVRs were assessed within the 200 Schaefer atlas regions. Interaction effects between regional Aβ-PET and TBI status on regional tau-PET were examined in linear regression models while adjusting for age, *APOE*ε4 carrier status, global tau-PET, and the time lag between the Aβ- and tau-PET scan. Subsequently, we identified ROIs showing significant interaction effects on both sides of the spectrum (FDR-corrected *p* < 0.05; either negative or positive interaction effects). We then averaged the Aβ-PET and tau-PET SUVRs of these significant ROIs and assessed the interaction between average Aβ-PET SUVR and TBI status on average tau-PET SUVR for negative (A) and positive (B) interaction effects separately, again using linear regression. The analyses were conducted on a sample of 98 individuals. Data points marked with circles represent CN individuals, while data points marked with triangles represent individuals with MCI. Cyan regression lines indicate no TBI, blue lines indicate mild TBI, and red lines indicate moderate/severe TBI. ^a,b^Mild TBI and moderate/severe TBI were significantly different from no TBI. Aβ = amyloid-β. AD = Alzheimer’s disease. *APOE* = apolipoprotein E. CN = cognitively normal. FDR = false discovery rate. MCI = mild cognitive impairment. PET = positron emission tomography. ROI = region of interest. SUVR = standardized uptake value ratio. TBI = traumatic brain injury.

**
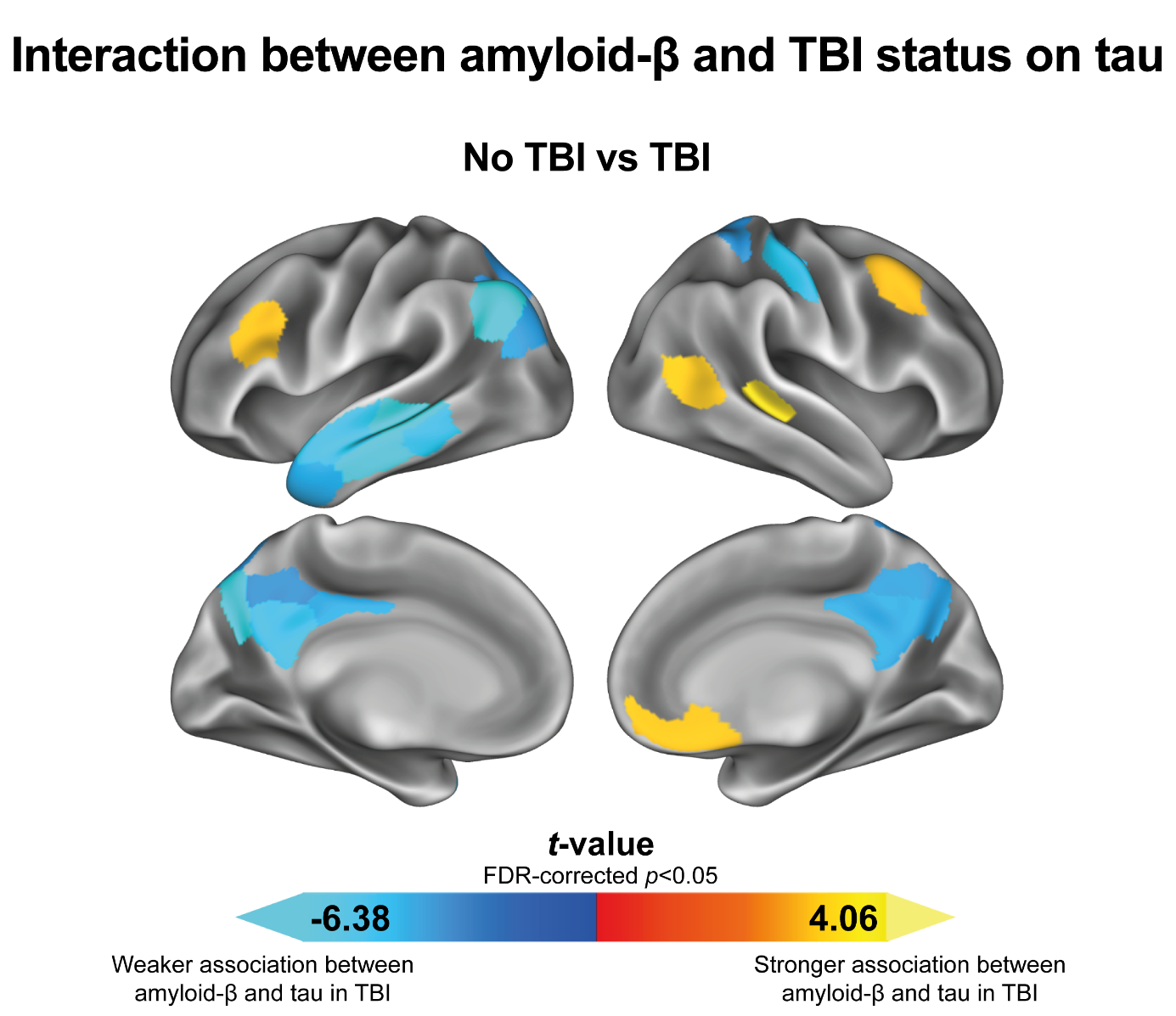
**

**Supplementary Fig. 6: Interaction between Aβ-PET and TBI status on tau-PET within the 200 Schaefer atlas regions.** Aβ- and tau-PET SUVRs were assessed within the 200 Schaefer atlas regions. Interaction effects were examined in linear regression models while adjusting for age, *APOE*ε4 carrier status, global tau-PET, and the time lag between the Aβ- and tau-PET scan. The analyses were conducted on a sample of 98 individuals. Only FDR-corrected significant group differences are shown. Positive *t*-values indicate stronger associations between Aβ-PET and tau-PET in the presence of a history of TBI, while negative *t*-values indicate the opposite. Aβ = amyloid-β. *APOE* = apolipoprotein E. FDR = false discovery rate. PET = positron emission tomography. SUVR = standardized uptake value ratio. TBI = traumatic brain injury.
